# Supplementary material for: A protocol for the Hearing impairment in Adults: A Longitudinal Outcomes Study (HALOS)
Source: PLoS One. 2023 Mar 16;18(3):e0283171. doi: 10.1371/journal.pone.0283171 (PMC10019733; doi:10.1371/journal.pone.0283171)
Supplement: S1 Appendix — (DOCX) [file pone.0283171.s001.docx]

Appendix 1. Semi-structured interview guide

*Icebreaker question*

1. Could you describe the hearing loss that you have (e.g., does it affect one or both ears, how severe is the hearing loss, how long have you had a hearing loss, does it run in the family)?

*Questions regarding patient experience of hearing health services*

1. Could you tell me about the treatment/s or services you have received or accessed specifically for your hearing loss (e.g., visiting GP/ audiologist and/or ENT, use hearing aid/cochlear implant, duration of use, any other treatment/rehabilitation)?
2. Could you describe the steps or the referral pathway you went through from finding out you have a hearing loss to receiving the treatment (hearing aid, cochlear implant and/or other) rehabilitation you needed?

- Detail of formal diagnosis and steps after, which health professionals referred/ involved etc.

1. Following your treatment/rehabilitation (hearing aid fitting/cochlear implantation), have you continued to receive adequate support from your hearing health service provider or any other healthcare professional? (e.g., regular follow-ups, support with adjusting to using your hearing device)

- Detail of any post-treatment support offered and accessed

1. On a scale, of 1 to 10 where 10 is very positive, how would you rate your experience throughout this hearing health service journey? Why did you give it this rating?
2. Based on your experience, how could the current hearing health service be improved or modified? Could any changes be made to make the process easier or the experience better?
3. Do you have any further comments regarding your experience or anything else we have spoken about today?
