# Supplementary material for: A protocol for the Hearing impairment in Adults: A Longitudinal Outcomes Study (HALOS)
Source: PLoS One. 2023 Mar 16;18(3):e0283171. doi: 10.1371/journal.pone.0283171 (PMC10019733; doi:10.1371/journal.pone.0283171)
Supplement: Attachment — Submitted filename: Response to reviewers.docx [file pone.0283171.s002.docx]

**Additional Editor Comments:**

**Comment 1a). Can authors please define the “baseline” for readers? I assume it is the time of recruitment, not the time of first using the hearing devices.**

Response 1a). Thank you for taking the time to review this manuscript. Yes, you are correct. The term “baseline” for this study does refer to the first recruitment time. We are recruiting people that are already current users of hearing devices, so it is not the first time using the hearing device. To ensure clarity in the paper, the use of the term “baseline” now includes in brackets “time of recruitment” at first mention in both the Abstract and main text.

**Comment 1b). Then period of hearing devices use at the baseline is vary between participants. How authors hope to manage that? This is applicable because concept of longitudinal design is based on outcomes likely to vary with time.**

Response 1b). Thank you, this is a very important point. At baseline, we ask participants when they started using a hearing aid and/or cochlear implant, therefore, the variation in duration of hearing device use can be adjusted for in subsequent multivariate analysis. This has now been specified in the ‘Statistical Analyses’ section of the manuscript, page 10, “Confounding factors e.g., age, sex, baseline pure-tone average thresholds, duration of hearing device use, will be included and adjusted for in the final models.”

**Comment 2). Cover letter says this is a pilot study, can I assume that is a typo because the manuscript is for a full study?**

Response 2a). Apologies for the typo. The title of the study in the cover letter has been amended to “A protocol for the Hearing impairment in Adults: a Longitudinal Outcomes Study (HALOS)”


**Reviewer 1 Comments:**

**Comment 1a). This is a description of a study protocol regarding hearing impairments in adults based on HALOS. The paper does not contain any results – not either preliminary results – but I think the ideas behind this upcoming study could be of interest for other researchers. My main comment is the structure of the protocol. I would have preferred that the paper was structured based on the 5 overall aims. So, for each of the 5 aims please describe the study population and the statistical methods used to investigate that specific question. It would help the reader to understand what you expect to do, and to better understand each of the specific aims – for instance aim 3 is unclear to me.**

Response 1a). Thank you to Reviewer 1 for taking the time to review this manuscript and provide suggestions for improvement before publication. We greatly appreciate the suggestion to improve the structure of the manuscript so there is a clearer link between the study aims and methodology. We feel that there would be some overlapping information if we restructured this protocol paper based on each of the 5 aims, therefore, we have now added sentences to state the relevant aims addressed under existing subheadings in the Methodology. Specifically, this includes:

Page 5. For the quantitative component, we have stated at the beginning of this section that *“Data from the quantitative component will be used to address the first three study aims, which are to evaluate the: 1. impacts of treating hearing loss on health (e.g., QoL, cognition, depression/mood, functional status), interpersonal relationships, education, and work; 2. differences in long-term outcomes within and between groups of hearing device users; 3. impact from the timing of audiological intervention and the effectiveness of earlier intervention on outcomes.”*

We have similarly linked these three aims to the follow-up data (page 11), stating that *“These longitudinal data… help contribute to achieving the first three study aims that were previously mentioned’.*

Page 9. For the qualitative component, we have stated at the beginning of this section that *“Data from the qualitative component will be used to address the fourth study aim, which is to better understand the healthcare journey for an adult with hearing loss, and identify the facilitators and barriers to accessing and using a hearing device.”*

Page 10. For the economic analyses, we have stated at the beginning of this section that *“The economic analysis will provide data to address the fifth study aim, which is to estimate the cost-effectiveness of early intervention through hearing aid(s) and/or cochlear implant(s) for hearing loss.”*

For the statistical analyses, we have included in brackets the relevant aims addressed for each type of analyses.

Page 9. *“To understand the complex relationships between treating hearing loss and health and social outcomes (i.e., Aim #1), structural equation modelling and path analyses will be conducted to examine direct and indirect pathways. To test for fixed effects for the two groups with consideration of the random effects from subjects (i.e., Aim #2), linear mixed effects regression models will be used. Differences within and between the two groups over time, as well as interaction effects between health and social outcomes will also be examined. Confounding factors (e.g., duration of hearing device use) will be included and adjusted for in the models. To understand the patterns, trajectories and profiling developmental pathways for hearing impairment (i.e., Aim #3), group-based trajectory modelling (GBTM) will be used.”*

**Comment 1b). Moreover, a language check would be preferable before publication (see for instance the fourth line of the introduction).**

Response 1b). Thank you for your suggestion. We have performed a thorough language check of the manuscript so that it meets the English standards for publication.
